# Assessment of the Feasibility of Using Noninvasive Wearable Biometric Monitoring Sensors to Detect Influenza and the Common Cold Before Symptom Onset

**DOI:** 10.1001/jamanetworkopen.2021.28534

**Published:** 2021-09-29

**Authors:** Emilia Grzesiak, Brinnae Bent, Micah T. McClain, Christopher W. Woods, Ephraim L. Tsalik, Bradly P. Nicholson, Timothy Veldman, Thomas W. Burke, Zoe Gardener, Emma Bergstrom, Ronald B. Turner, Christopher Chiu, P. Murali Doraiswamy, Alfred Hero, Ricardo Henao, Geoffrey S. Ginsburg, Jessilyn Dunn

**Affiliations:** 1 Biomedical Engineering Department, Duke University, Durham, North Carolina; 2 Duke Center for Applied Genomics and Precision Medicine, Duke University Medical Center, Durham, North Carolina; 3 Durham Veterans Affairs Medical Center, Durham, North Carolina; 4 Department of Medicine, Duke Global Health Institute, Durham, North Carolina; 5 Department of Infectious Disease, Imperial College London, London, United Kingdom; 6 Department of Pediatrics, University of Virginia School of Medicine, Charlottesville; 7 Department of Psychiatry, Duke University School of Medicine, Durham, North Carolina; 8 Department of Medicine, Duke University School of Medicine, Durham, North Carolina; 9 Department of Electrical Engineering and Computer Science, University of Michigan, Ann Arbor; 10 Department of Biostatistics and Bioinformatics, Duke University Medical Center, Durham, North Carolina

## Abstract

**Importance:**

Currently, there are no presymptomatic screening methods to identify individuals infected with a respiratory virus to prevent disease spread and to predict their trajectory for resource allocation.

**Objective:**

To evaluate the feasibility of using noninvasive, wrist-worn wearable biometric monitoring sensors to detect presymptomatic viral infection after exposure and predict infection severity in patients exposed to H1N1 influenza or human rhinovirus.

**Design, Setting, and Participants:**

The cohort H1N1 viral challenge study was conducted during 2018; data were collected from September 11, 2017, to May 4, 2018. The cohort rhinovirus challenge study was conducted during 2015; data were collected from September 14 to 21, 2015. A total of 39 adult participants were recruited for the H1N1 challenge study, and 24 adult participants were recruited for the rhinovirus challenge study. Exclusion criteria for both challenges included chronic respiratory illness and high levels of serum antibodies. Participants in the H1N1 challenge study were isolated in a clinic for a minimum of 8 days after inoculation. The rhinovirus challenge took place on a college campus, and participants were not isolated.

**Exposures:**

Participants in the H1N1 challenge study were inoculated via intranasal drops of diluted influenza A/California/03/09 (H1N1) virus with a mean count of 10^6^ using the median tissue culture infectious dose (TCID50) assay. Participants in the rhinovirus challenge study were inoculated via intranasal drops of diluted human rhinovirus strain type 16 with a count of 100 using the TCID50 assay.

**Main Outcomes and Measures:**

The primary outcome measures included cross-validated performance metrics of random forest models to screen for presymptomatic infection and predict infection severity, including accuracy, precision, sensitivity, specificity, F1 score, and area under the receiver operating characteristic curve (AUC).

**Results:**

A total of 31 participants with H1N1 (24 men [77.4%]; mean [SD] age, 34.7 [12.3] years) and 18 participants with rhinovirus (11 men [61.1%]; mean [SD] age, 21.7 [3.1] years) were included in the analysis after data preprocessing. Separate H1N1 and rhinovirus detection models, using only data on wearble devices as input, were able to distinguish between infection and noninfection with accuracies of up to 92% for H1N1 (90% precision, 90% sensitivity, 93% specificity, and 90% F1 score, 0.85 [95% CI, 0.70-1.00] AUC) and 88% for rhinovirus (100% precision, 78% sensitivity, 100% specificity, 88% F1 score, and 0.96 [95% CI, 0.85-1.00] AUC). The infection severity prediction model was able to distinguish between mild and moderate infection 24 hours prior to symptom onset with an accuracy of 90% for H1N1 (88% precision, 88% sensitivity, 92% specificity, 88% F1 score, and 0.88 [95% CI, 0.72-1.00] AUC) and 89% for rhinovirus (100% precision, 75% sensitivity, 100% specificity, 86% F1 score, and 0.95 [95% CI, 0.79-1.00] AUC).

**Conclusions and Relevance:**

This cohort study suggests that the use of a noninvasive, wrist-worn wearable device to predict an individual’s response to viral exposure prior to symptoms is feasible. Harnessing this technology would support early interventions to limit presymptomatic spread of viral respiratory infections, which is timely in the era of COVID-19.

## Key Points

**Question:**

Can noninvasive, wrist-worn wearable devices detect acute viral respiratory infection and predict infection severity before symptom onset?

**Findings:**

In a cohort study of 31 participants inoculated with H1N1 and 18 participants with rhinovirus, infection detection and severity prediction models trained using data on wearable devices were able to distinguish between infection and noninfection with 92% accuracy for H1N1 and 88% accuracy for rhinovirus and were able to distinguish between mild and moderate infection 24 hours prior to symptom onset with 90% accuracy for H1N1 and 89% accuracy for rhinovirus.

**Meaning:**

This study suggests that the use of wearable devices to identify individuals with presymptomatic acute viral respiratory infection is feasible; because wearable devices are common in the general population, using them for infection screening may help limit the spread of contagion.

## Teaser

This cohort study evaluates the feasibility of using noninvasive, wrist-worn wearable devices to detect presymptomatic viral infection after exposure and predict infection severity in patients exposed to H1N1 influenza or human rhinovirus.

## Introduction

Approximately 9% of the world is infected with influenza annually, resulting in 3 million to 5 million severe cases and 300 000 to 500 000 deaths per year.^1^ Adults are infected with approximately 4 to 6 common colds per year, and children are infected with approximately 6 to 8 common colds per year, with more than half of infections caused by human rhinoviruses (RVs).^2,3^ Given the highly infectious nature of respiratory viruses and their variable incubation periods, infections are often transmitted unwittingly in a manner that results in community spread, especially as no presymptomatic screening methods currently exist to identify respiratory viral diseases.^4,5^ With the increasing emergence of novel viruses, such as SARS-CoV-2,^6^ it is critical to quickly identify and isolate contagious carriers of a virus, including presymptomatic and asymptomatic individuals, at the population level to minimize viral spread and associated severe health outcomes.

Wearable biometric monitoring sensors (hereafter referred to as *wearables*) have been shown to be useful in detecting infections before symptoms occur.^7,8,9^ Low-cost and accessible technologies that record physiologic measurements can empower underserved groups with new digital biomarkers.^8,10,11,12^ Digital biomarkers are digitally collected data that are transformed into indicators of health and disease.^13,14^ For example, resting heart rate, heart rate variability, accelerometry, electrodermal skin activity, and skin temperature can indicate a person’s infection status^8,9,15,16,17,18,19,20,21,22,23,24,25,26,27^ or predict if and when a person will become infected after exposure.^7^ Therefore, detecting abnormal biosignals using wearables could be the first step in identifying infections before symptom onset.^8^

**Figure 1.**
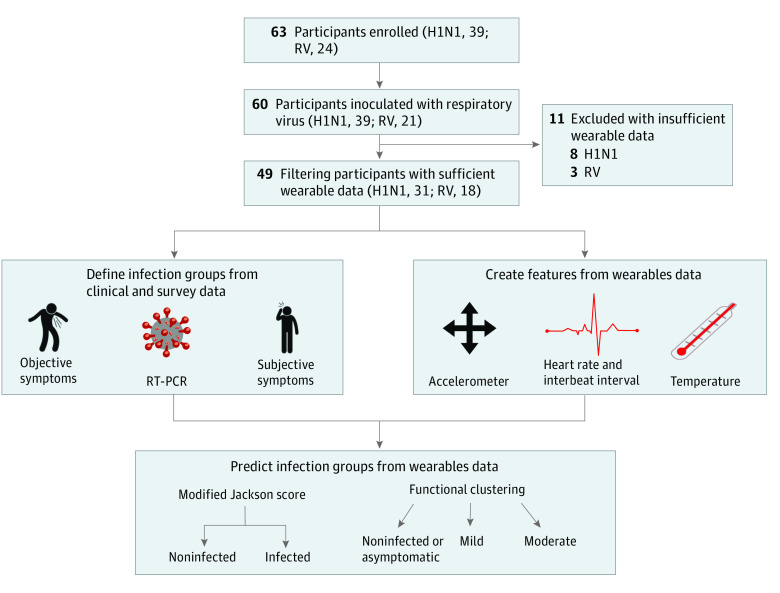
Flow Diagram and Graphical Abstract of Study RV indicates rhinovirus; RT-PCR, reverse transcription polymerase chain reaction; and wearables, wearable biometric monitoring sensors.

Here, we developed digital biomarker models for early detection of infection and severity prediction after pathogen exposure but before symptoms develop (Figure 1). Our results highlight the opportunity for the identification of early presymptomatic or asymptomatic infection that may support individual treatment decisions and public health interventions to limit the spread of viral infections.

## Methods

### Study Population

A total of 39 participants (12 women and 27 men; aged 18-55 years; mean [SD] age, 36.2 [11.8] years; 2 [5.1%] Black, 6 [15.4%] Asian, 25 [64.1%] White, 2 [5.1%] ≥2 race categories [1 (2.6%) White and Caribbean; 1 (2.6%) mixed/other category], and 4 [10.3%] did not fall into any of the ethnic groups listed, so they identified as “all other ethnic groups”) were recruited for the H1N1 influenza challenge study. Data were collected from September 11, 2017, to May 4, 2018. The influenza challenge study was reviewed and approved by the institutional review board at Duke University and the London-Fulham Research Ethics Committee. Written informed consent was obtained from all participants. A total of 24 participants (8 women and 16 men; aged 20-34 years; mean [SD] age, 22 [3.1] years; and 1 [4.2%] Black, 6 [25.0%] Asian, and 15 [62.5%] White, including 3 [12.5%] Hispanic or Latinx, 1 [4.2%] White and Black mixed, and 1 [4.2%] unknown) were recruited for the RV challenge study. Data were collected from September 14 to 21, 2015. The RV challenge study was reviewed and approved by the institutional review board at Duke University and the University of Virginia. Written informed consent was obtained from all participants. This study followed the Strengthening the Reporting of Observational Studies in Epidemiology (STROBE) and the Transparent Reporting of a Multivariable Prediction Model for Individual Prognosis or Diagnosis (TRIPOD) reporting guidelines.

### Exclusion Criteria

Exclusion criteria for the influenza challenge included current pregnancy, breastfeeding, or smoking; history of chronic respiratory, allergy, or other significant illness; recent upper respiratory tract infection; nose abnormalities; or immunocompromised status. Participants were screened for high levels of serum antibodies against the challenge strain by hemagglutination inhibition assay (titers >1:10 excluded).^7^ Exclusion criteria for the RV challenge included pregnancy; chronic respiratory illness; high blood pressure; history of tobacco, drug, or alcohol use; and serum antibody titers more than 1:4.

### Study Protocol

Participants in the H1N1 challenge study wore the E4 wristband (Empatica Inc) 1 day before and 11 days after the inoculation on the morning of day 2, before clinical discharge. The E4 wristband measures heart rate, skin temperature, electrodermal activity, and movement. Participants were inoculated via intranasal drops of the diluted influenza A/California/03/09 (H1N1) virus with a mean count of 10^6^ using the median tissue culture infectious dose (TCID50) assay in 1-mL phosphate-buffered saline and were isolated for at least 8 days after inoculation after negative results of a nasal lavage polymerase chain reaction test.^7^ We defined symptoms as either observable events (fever, stuffy nose, runny nose, sneezing, coughing, shortness of breath, hoarseness, diarrhea, and wheezy chest) or unobservable events (muscle soreness, fatigue, headache, ear pain, throat discomfort, chest pain, chills, malaise, and itchy eyes).^28^ Viral shedding was quantified by nasal lavage polymerase chain reaction each morning, and symptoms were self-reported twice daily.

Participants in the RV challenge study wore the E4 wristband for 4 days before and 5 days after inoculation, which occurred in the afternoon (1-5 pm) via intranasal drops of diluted human RV strain type 16 with a count of 100 using the TCID50 assay in 1 mL of lactated Ringer solution. Participants underwent daily nasal lavage, and the symptoms were reported as previously described. Participants lived on a college campus and were not isolated.

### Data Preparation and Preprocessing

**Figure 2.**
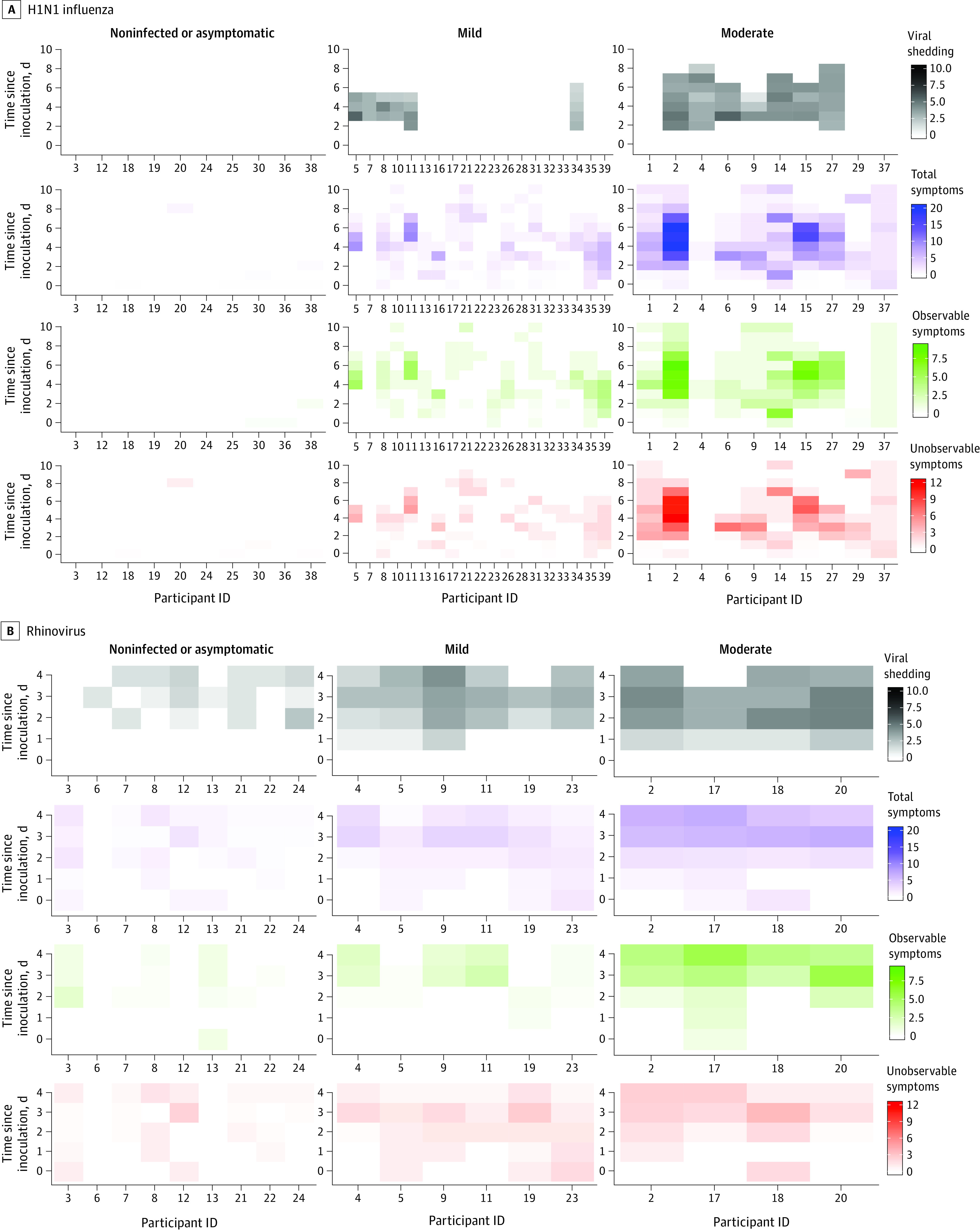
Infection Severity Categorization Based on Functional Clustering of Daily Symptoms and Shedding A, H1N1 influenza. B, Rhinovirus. ID indicates identification.

We grouped individuals by infection similarity (Figure 2) using data-driven methods based on infection severity (asymptomatic or noninfected [AON], mild, or moderate signs of infection) and trajectory (early, middle, or late signs of infection). Multivariate functional clustering (bayesian information criteria loss function) was done on 3 daily aggregate measurements: observable symptoms, unobservable symptoms, and viral shedding.^29,30^ Clinical infection groups were determined by previous definitions of symptomatic (modified Jackson symptom score >5 within first 5 days of inoculation) and viral shedders (>2 days of shedding).^31,32,33,34^ Participants who were positive in one criterion but not the other were excluded from further analysis in the clinical groupings. For both infection groupings, we defined symptom onset as the first day of a 2-day period in which the symptom score was at least 2 points.^32^

Mean (SD) and median values of heart rate, skin temperature, and accelerometry were calculated every minute from baseline to 60 hours after inoculation. If several preinoculation days were present, then the baseline was defined as the mean value of each wearable metric at the same time of day. A total of 8 and 3 participants were removed from the H1N1 and RV analyses, respectively, owing to lack of sufficient data caused by nonwear, miswear, or device errors, which were detected following the methods of She et al.^7^

Resting heart rate and temperature were defined by a 5-minute median accelerometer cutoff determined from the baseline day’s data.^35^ For each 12-hour interval, several interbeat interval features were calculated using the 5-minute rolling mean with baseline subtraction: mean heart rate variability, median heart rate variability, number of successive N-N intervals that differ by more than 50 milliseconds, percentage of N-N intervals that differ by more than 50 milliseconds, SD of N-N intervals, and root mean square of successive R-R interval differences (eTable 1 in the Supplement).^35^ To account for circadian effects, model features were calculated as the difference between preinoculation and postinoculation summary metrics occurring at the same 1-hour clock time of day (eTable 1 and eFigure 1A in the Supplement).^36^

Models predicting infection further in time after inoculation included progressively more features (9 features added for each 12-hour block; eFigure 1B in the Supplement). Performance relative to symptom onset was calculated by differencing the time after inoculation from the median symptom onset of each viral challenge. The resulting feature set consisted of 40 features calculated from 9 delta summary wearable metrics generated from five 12-hour intervals. Forward stepwise selection simultaneously tuned models and performed feature selection to prevent overfitting.^37^

### Machine Learning Models

Bootstrapped binary or multiclass random forest classifiers were built using Python Scikit-learn and validated using leave-one-person-out cross-validation (trees = 1000).^12,37,38^ This procedure was repeated for every 12-hour period feature set that was added to a model (eFigure 1B in the Supplement).

### Statistical Analysis

Evaluation metrics of the models included accuracy, precision, sensitivity, specificity, F1 score, and area under the receiver operating characteristic curve (AUC).^39^ The primary metric of model success was accuracy. For multiclass models, the weighted mean value for each metric was recorded. For binary models, receiver operating characteristic curves were derived from the predicted class probabilities of an input sample, and the resulting AUC and 95% CI were reported.

## Results

### Study Summary

The data were generated as part of 2 large challenge studies involving nasal lavage inoculation of human volunteers with either influenza (H1N1) or human RV. For the influenza prediction models, 31 participants were included in the analysis after data preprocessing (7 women and 24 men; aged 18-55 years; mean [SD] age, 34.7 [12.3] years; and 5 [16.1%] Asian, 21 [67.7%] White, 1 [3.2%] mixed/other category, and 4 [12.9%] did not fall into any of the ethnic groups listed, so they identified as “all other ethnic groups”). For the RV prediction models, 18 participants were included in the analysis after preprocessing (7 women and 11 men; aged 20-33 years; mean [SD] age, 21.7 [3.1] years; and 2 [11.1%] Asian, 2 [11.1%] Black, and 14 [77.8%] White, including 3 [16.7%] Hispanic or Latinx). The primary demographic difference between the 2 viral challenges was that the H1N1 group contained a wider age range and a higher mean age of participants (eTable 2A and B in the Supplement).

Functional clustering indicated that there were 3 distinct classes of infection status that, on visual inspection, roughly equated to (1) AON, (2) mild, and (3) moderate (Figure 2). Based on this clustering, we defined the data-driven “infected” group as the combined mild and moderate classes and the “noninfected” group as the AON class. All clinically driven labels of infected vs noninfected were perfectly replicated by the data-driven groupings for the RV challenge but not for the H1N1 challenge.^40^

### Prediction of Infection After Exposure Using Wearables

**Figure 3.**
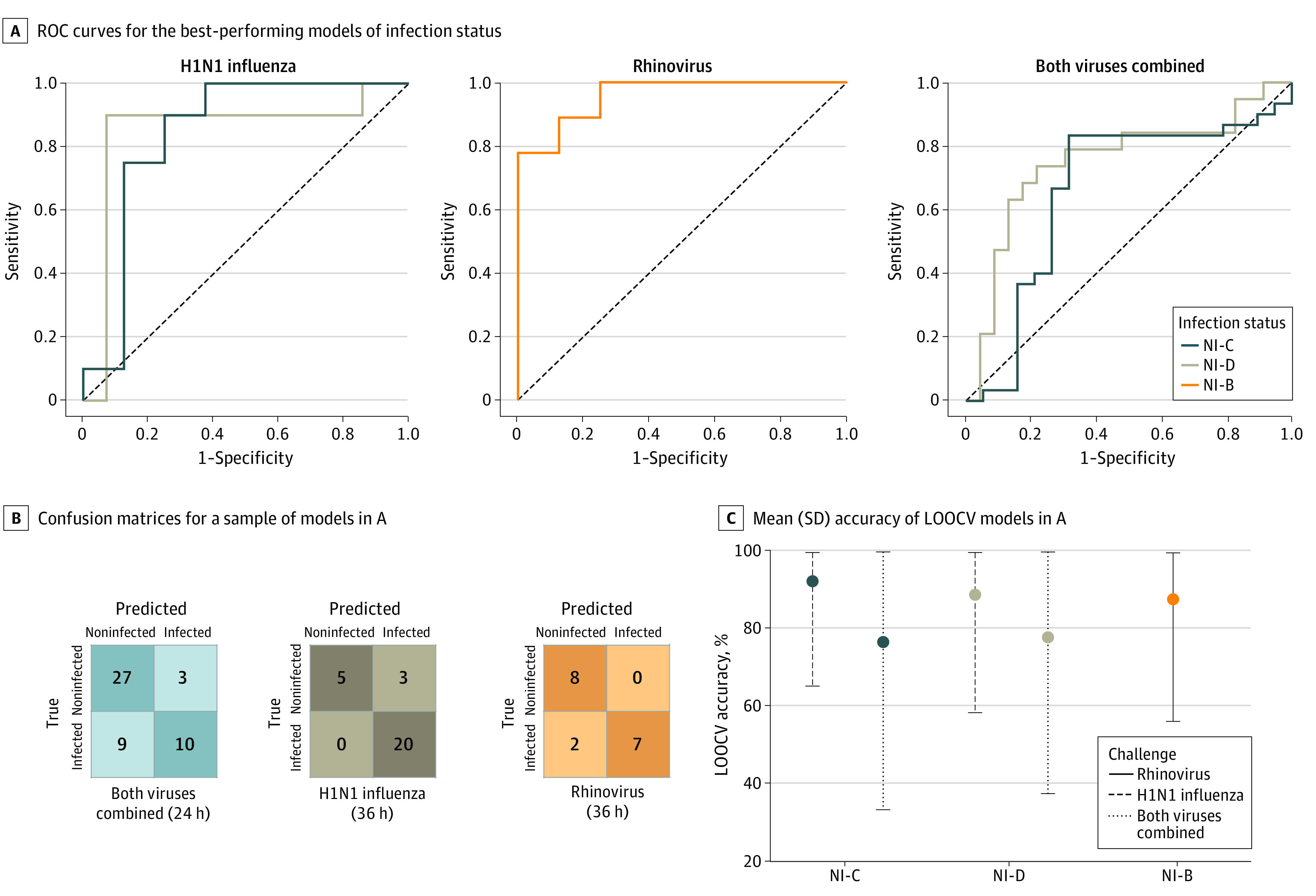
Performance Metrics of the Best-Performing Models for Predicting Infection Status (Infected vs Noninfected) A, Receiver operating characteristic (ROC) curves for the best-performing models of infection status for the H1N1 influenza, rhinovirus, and combined virus challenges. B, Confusion matrices for a sample of models in A. C, Mean (SD) accuracy of leave-one-out, cross-validated (LOOCV) models in A. NI-B indicates noninfected vs infected, both; NI-C, noninfected vs infected, clinical; and NI-D, noninfected vs infected, data driven.

**Figure 4.**
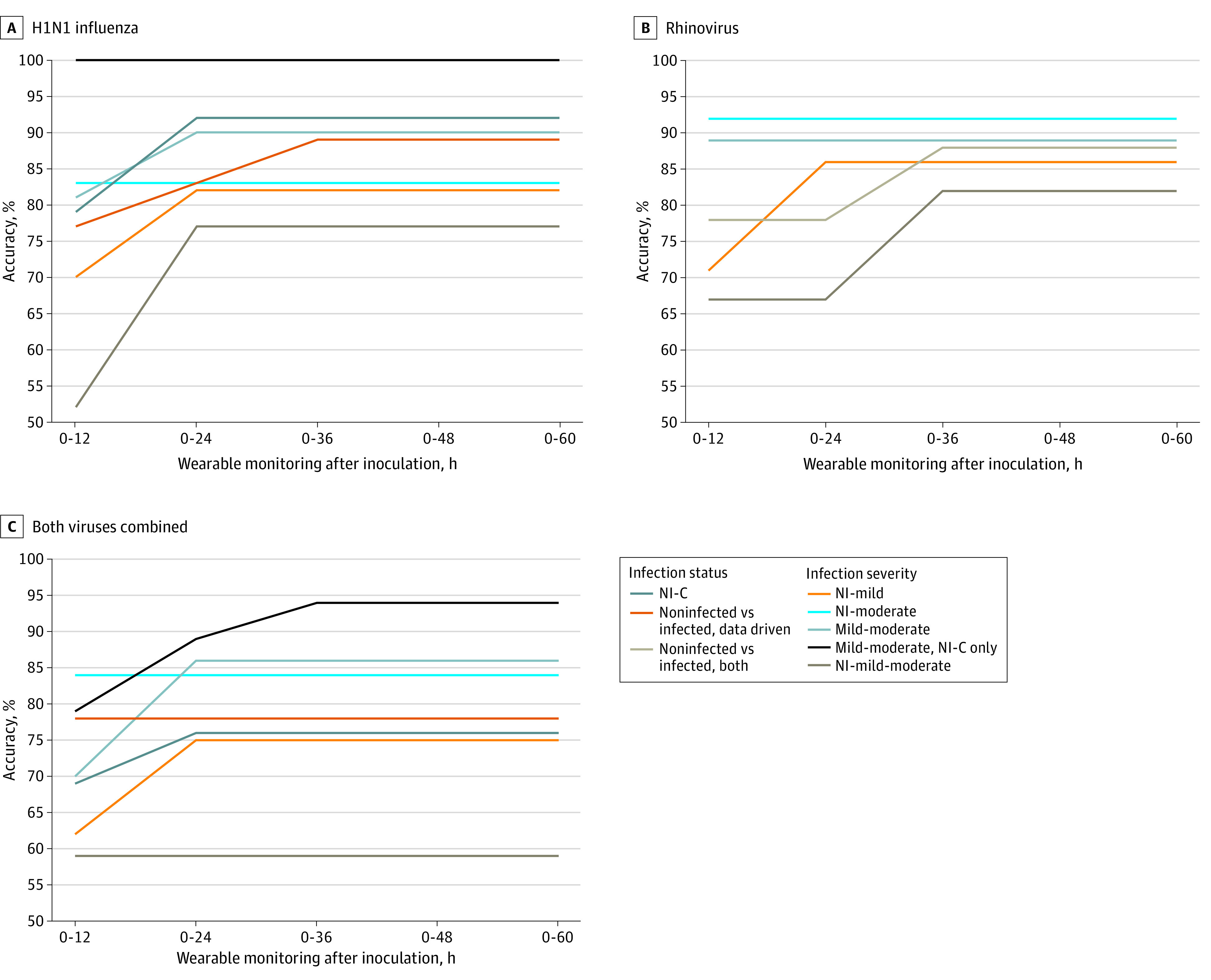
Model Accuracy Over Time Across All Viral Challenges, Infectious Status Groupings, and Infection Severity Groupings A, H1N1 influenza. B, Rhinovirus. C, Both viruses combined. Mild-moderate, mild to moderate; NI-C, noninfected vs infected, clinical; NI-mild, noninfected vs infected, mild; NI-mild-moderate, noninfected vs infected, mild to moderate; NI-moderate, noninfected vs infected, moderate; and wearables, wearable biometric monitoring sensors.

We developed 25 binary, random forest classification models to predict infection vs noninfection using features derived from wearables. Each model covered a different time period after inoculation or used a different definition of infected vs noninfected. For infected participants in the H1N1 challenge, the median symptom onset after inoculation was 48 hours (range, 9-96 hours). At 36 hours after inoculation, models predicting the data-driven groupings from the H1N1 challenge reached an accuracy of 89% (87% precision, 100% sensitivity, 63% specificity, 93% F1 score, and 0.84 [95% CI, 0.60-1.00] AUC). Because 7 participants were either symptomatic nonshedders (n = 6) or AON shedders (n = 1), the clinically driven H1N1 infection groupings had 7 fewer observations than the data-driven groupings. Models predicting the clinically driven groupings for H1N1 reached an accuracy of 79% (72% precision, 80% sensitivity, 79% specificity, 76% F1 score, and 0.68 [95% CI, 0.46-0.89] AUC) within 12 hours after inoculation and an accuracy of 92% (90% precision, 90% sensitivity, 93% specificity, 90% F1 score, and 0.85 [95% CI, 0.70-1.00] AUC) within 24 hours after inoculation. Regardless of whether the data-driven or clinically driven grouping method was used, we could assess whether or not a participant was infected with H1N1 between 24 and 36 hours before symptom onset (Figure 3A-C; Figure 4A; and eFigure 2 and eTable 3 in the Supplement).

The median symptom onset for RV was 36 hours after inoculation (range, 24-36 hours). The models predicting whether or not a participant was infected with RV achieved an early accuracy of 78% (78% precision, 78% sensitivity, 78% specificity, 78% F1 score, and 0.77 [95% CI, 0.54-0.99] AUC) at 12 hours after inoculation. This time point corresponded to 24 hours prior to symptom onset. Model performance peaked at the time of symptom onset, which was 36 hours after inoculation, with an accuracy of 88% at the same time as symptom onset (100% precision, 78% sensitivity, 100% specificity, 88% F1 score, and 0.96 [95% CI, 0.85-1.00] AUC) (Figure 3A-C; Figure 4B; and eFigure 2 and eTable 3 in the Supplement).

When both viral challenges were combined, models predicting the data-driven infection groupings reached an early accuracy of 78% (81% precision, 83% sensitivity, 68% specificity, 82% F1 score, and 0.66 [95% CI, 0.50-0.82] AUC) at 12 hours after inoculation. The models predicting clinically driven infection groupings reached an accuracy of 76% (76% precision, 68% sensitivity, 83% specificity, 72% F1 score, and 0.75 [95% CI, 0.60-0.90] AUC) at 24 hours after inoculation (Figure 3A-C; Figure 4C; and eFigure 2 and eTable 3 in the Supplement).

### Prediction of Infection Severity Prior to Symptom Onset Using Wearables

Infection severity was defined as (1) AON, (2) mild, or (3) moderate based on the data-driven functional clustering results (Figure 2). We developed 66 binary and multiclass random forest models to predict class membership using features derived from the wearables for different time periods after inoculation. After automated feature selection, all 41 of the single viral challenge models included only 1 to 3 of the 9 to 45 possible features per model (eFigure 4A and B in the Supplement). Interbeat interval features were retained in every model, and resting heart rate features were present in almost half (47.4% [9 of 19]) of the models (eTable 1 in the Supplement).

**Figure 5.**
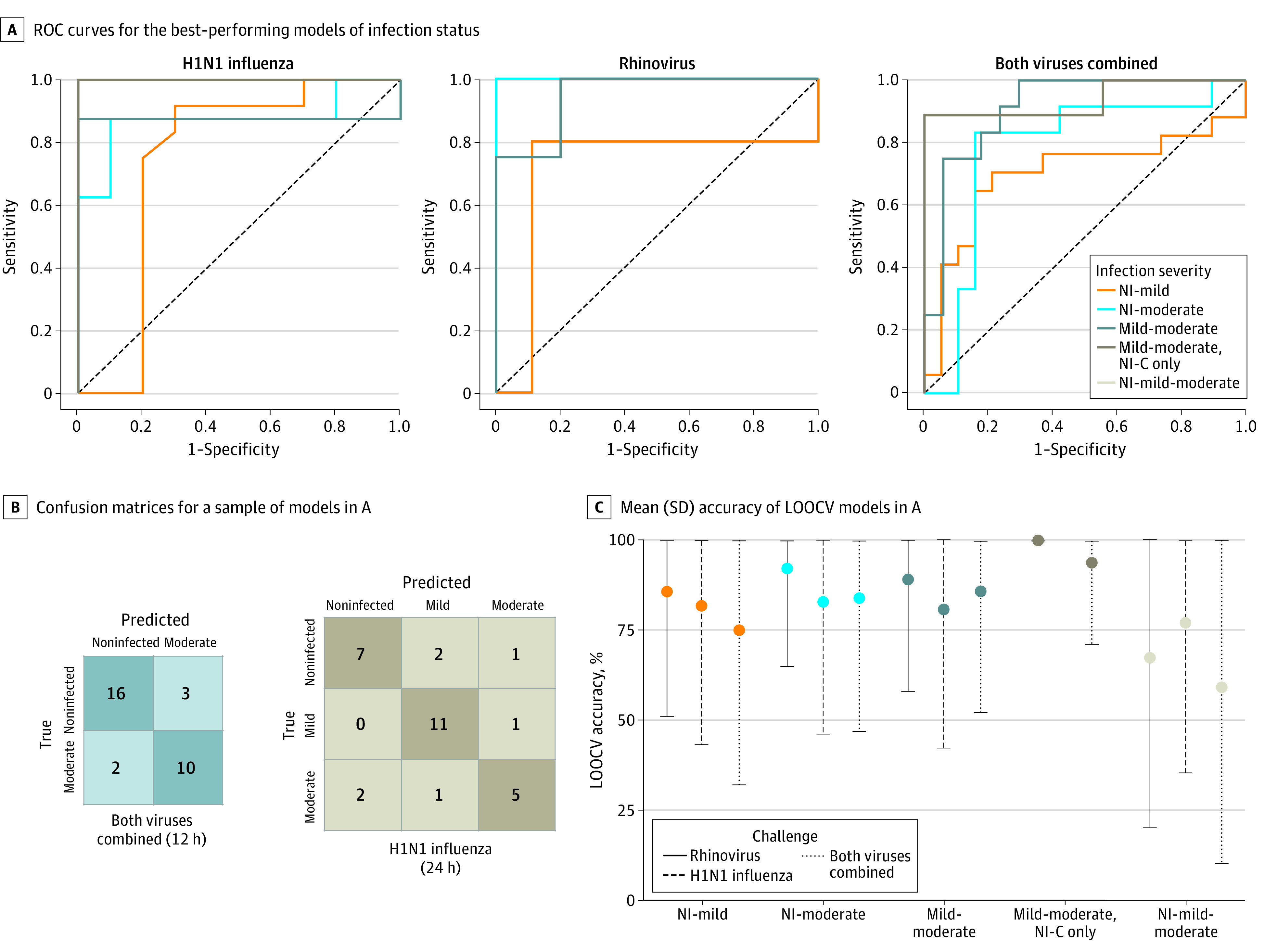
Performance Metrics of the Best-Performing Models for Predicting Infection Severity A, Receiver operating characteristic (ROC) curves for the best-performing models of infection status for the H1N1 influenza, rhinovirus, and combined virus challenges. B, Confusion matrices for a sample of models in A. C, Mean (SD) accuracy of leave-one-out, cross-validated (LOOCV) models in A. Mild-moderate indicates mild to moderate; NI-C, noninfected vs infected, clinical; NI-mild, noninfected vs infected, mild; NI-mild-moderate, noninfected vs infected, mild to moderate; and NI-moderate, noninfected vs infected, moderate.

At 12 hours after inoculation, the binary classification model predicting the future development of AON vs moderate H1N1 achieved 83% accuracy (78% precision, 88% sensitivity, 80% specificity, 82% F1 score, and0.88 [95% CI, 0.71-1.00] AUC). For RV, the model predicting the future development of AON vs moderate infection reached 92% accuracy (80% precision, 100% sensitivity, 89% specificity, 89% F1 score, and 1.00 [95% CI, 1.00-1.00] AUC). For both viruses combined, the model predicting the future development of AON vs moderate infection peaked at 84% accuracy (77% precision, 83% sensitivity, 84% specificity, 80% F1 score, and 0.78 [95% CI, 0.61-0.94] AUC) at 12 hours after inoculation (Figure 4A-C; Figure 5A-C; and eFigure 3, eTable 4, and eTable 5 in the Supplement).

Of the binary classification models for both viral challenge studies, we found that the AON vs moderate models achieved the highest accuracy and AUC toward predicting infection severity prior to symptom onset. This finding was expected given that these were the 2 most divergent classes of infection severity. At 12 hours after inoculation, the model predicting mild vs moderate H1N1 distinguished between the 2 symptomatic groups with 81% accuracy (75% precision, 75% sensitivity, 85% specificity, 75% F1 score, and 0.86 [95% CI, 0.69-1.00] AUC). By 24 hours after inoculation, this model achieved 90% accuracy (88% precision, 88% sensitivity, 92% specificity, 88% F1 score, and 0.88 [95% CI, 0.72-1.00] AUC). After excluding H1N1 challenge participants in the mild and moderate classes who did not have an infection per the clinically driven definition, the model predicting mild vs moderate H1N1 achieved 100% accuracy (100% precision, 100% sensitivity, 100% specificity, 100% F1 score, and 1.00 [95% CI, 1.00-1.00] AUC). By 24 hours after inoculation, the infection severity prediction model was able to distinguish between mild and moderate infection with an accuracy of 89% for RV (100% precision, 75% sensitivity, 100% specificity, 86% F1 score, and 0.95 [95% CI, 0.79-1.00] AUC). The model predicting mild vs moderate illness for both viruses combined distinguished between the 2 symptomatic groups with an accuracy of 86% (90% precision, 75% sensitivity, 94% specificity, 82% F1 score, and 0.91 [95% CI, 0.80-1.00] AUC). After excluding H1N1 challenge participants in the mild and moderate classes who did not have an infection per the clinically driven definition, the model predicting mild vs moderate illness for both viruses combined reached an accuracy of 94% (100% precision, 89% sensitivity, 100% specificity, 94% F1 score, and 0.94 [95% CI, 0.82-1.00] AUC) (Figure 4B; Figure 5A and C; eFigure 3, eTable 4, and eTable 5 in the Supplement). Receiving operator characteristic curves for both viral challenge studies (Figure 5A) demonstrated that the model predicting development of AON vs moderate illness and the model predicting development of mild vs moderate illness yielded higher discriminative ability than the model predicting AON vs mild illness.

The multiclass models were built to predict both infection status and infection severity per the data-driven definitions. We found that the highest performing multiclass models (predicting development of AON vs mild vs moderate illness) reached 77% accuracy for H1N1 (24 hours after inoculation; 76% precision, 77% sensitivity, 88% specificity, and 76% F1 score) and 82% accuracy for RV (36 hours after inoculation; 85% precision, 82% sensitivity, 88% specificity, and 82% F1 score) (Figure 4B; Figure 5B and C; eFigure 2 and eTable 4 in the Supplement).

## Discussion

The aim of this work was to evaluate a novel and scalable approach to identify whether or not a person will develop an infection after virus exposure and to predict eventual disease severity using noninvasive, wrist-worn wearables. The approach was tested using 2 viral challenge studies with influenza H1N1, human RV, or both viruses combined. This study shows that it is feasible to use wearable data to predict infection status and infection severity 12 to 36 hours before symptom onset, with most of our models reaching greater than 80% accuracy. Presymptomatic detection of respiratory viral infection and infection severity prediction may enable better medical resource allocation, early quarantine, and more effective prophylactic measures. Our results show that an accuracy plateau occurred in the 12- to 24-hour period after inoculation for 24 of 25 infection detection models (96.0%) and for 64 of 66 infection severity models (97.0%). This finding indicates that the most critical of the physiologic changes that occur in response to viral inoculation and that predict pending illness severity occurred within 12 to 24 hours after exposure.

Two factors associated with model accuracy are (1) knowledge of the exact time and dosage of inoculation and (2) the high-fidelity measurements of the research-grade wearable that enable intricate feature engineering, neither of which are possible in existing observational studies using consumer-grade devices. Because the outcome labeling is robust and accurate, there is a significant reduction in noise that would be present in an observational study.^41^ The participants in both studies experienced clinically mild disease, so the physiologic changes in patients with severe disease outcomes would likely be even more extreme and therefore easier to detect. The timing of the models’ detection and severity prediction is particularly relevant to current work aimed at early detection of COVID-19 from smartwatches, as presymptomatic and asymptomatic spread are significant contributors to the SARS-CoV-2 pandemic.^9,20,21,22,23,24,26,42,43,44,45^ The most important features for predicting infection severity were resting heart rate and mean heart rate variability. Thus, our model could be extensible to commercial wearables, which are used by 21% of US adults, for population-level detection of respiratory viral infections.^46,47^

Several factors may be associated with the higher accuracy of the RV severity models compared with the H1N1 severity models, including the longer RV baseline period (4 days vs 1 day) and the morning vs afternoon inoculation time that may include circadian effects. This possibility was addressed in part by calculating the differences between baseline and postinoculation only from measurements taken at the same times of day. The same influenza challenge data were recently used to predict viral shedding timing, with an AUC of 0.758 using heart rate during sleep as a model feature.^7^ Nighttime and early morning biometric measurements are potentially more useful than daytime measurements owing to their increased consistency, which should be explored further in future studies.^8,36^

### Limitations

This study has some limitations. It focuses on 2 common respiratory viruses in a fairly small population. Expanding the data set to include larger and more diverse populations and other types of viruses will be necessary to demonstrate the broad applicability of these findings. Inclusion of negative control groups (ie, participants with no pathogen exposure and those with conditions that masquerade as infections [eg, asthma or allergies]) would further improve the work.

## Conclusions

This study suggests that routine physiologic monitoring using common wearable devices may identify impending viral infection before symptoms develop. The ability to identify individuals during this critical early phase, when many may be spreading the virus without knowing it, and when therapies (if available) and public health interventions are most likely to be efficacious, may have a wide-ranging effect. In the midst of the global SARS-CoV-2 pandemic, the need for novel approaches like this has never been more apparent, and future work to validate these findings in individuals with other respiratory infections, such as COVID-19, may be critical given the highly variable and potentially severe or even fatal presentation of SARS-CoV-2 infection. The ability to detect infection early, predict how an infection will change over time, and determine when health changes occur that require clinical care may improve resource allocation and save lives.

## Notes

Supplement. **eTable 1.** Features Used in Random Forest Models **eFigure 1.** Feature Sets for Every Model **eFigure 2.** Confusion Matrices for Best Performing Model Across Viral Challenges and Infection Status Comparisons **eFigure 3.** Confusion Matrices for Best Performing Model Across Viral Challenges and Infection Severity Comparisons **eFigure 4.** Relative Feature Importance for Best Performing Model for Each Viral Challenge, Infection Status Grouping, and Infection Severity Grouping **eTable 2.** Racial Demographics, Sex, and Median Age Across Infection Severity Groups for H1N1 Influenza Viral Challenge and Rhinovirus Viral Challenge **eTable 3.** Mean Accuracy, Precision, Sensitivity, F1-Score, AUC of Every Infection Status Model Tested Across Viral Challenges, Number of Hours Post-Inoculation, and Infection Severity Comparisons **eTable 4.** Mean Accuracy, Precision, Sensitivity, Specificity, F1-Score, AUC of Every Infection Severity Model Tested Across Individual Viral Challenges, Number of Hours Post-Inoculation, and Infection Severity Comparisons **eTable 5.** Mean Accuracy, Precision, Sensitivity, Specificity, F1-Score, AUC of Every Infection Severity Model Tested Across Combined Viral Challenges, Number of Hours Post-Inoculation, and Infection Severity Comparisons
