# Supplementary material for: Assessment of the Feasibility of Using Noninvasive Wearable Biometric Monitoring Sensors to Detect Influenza and the Common Cold Before Symptom Onset
Source: JAMA Netw Open. 2021 Sep 29;4(9):e2128534. doi: 10.1001/jamanetworkopen.2021.28534 (PMC8482058; doi:10.1001/jamanetworkopen.2021.28534)
Supplement: Supplement. — eTable 1. Features Used in Random Forest Models eFigure 1. Feature Sets for Every Model eFigure 2. Confusion Matrices for Best Performing Model Across Viral Challenges and Infection Status Comparisons eFigure 3. Confusion Matrices for Best Performing Model Across Viral Challenges and Infection Severity Comparisons eFigure 4. Relative Feature Importance for Best Performing Model for Each Viral Challenge, Infection Status Grouping, and Infection Severity Grouping eTable 2. Racial Demographics, Sex, and Median Age Across Infection Severity Groups for H1N1 Influenza Viral Challenge and Rhinovirus Viral Challenge eTable 3. Mean Accuracy, Precision, Sensitivity, F1-Score, AUC of Every Infection Status Model Tested Across Viral Challenges, Number of Hours Post-Inoculation, and Infection Severity Comparisons eTable 4. Mean Accuracy, Precision, Sensitivity, Specificity, F1-Score, AUC of Every Infection Severity Model Tested Across Individual Viral Challenges, Number of Hours Post-Inoculation, and Infection Severity Comparisons eTable 5. Mean Accuracy, Precision, Sensitivity, Specificity, F1-Score, AUC of Every Infection Severity Model Tested Across Combined Viral Challenges, Number of Hours Post-Inoculation, and Infection Severity Comparisons [file jamanetwopen-e2128534-s001.pdf]

Supplementary Online Content

Grzesiak E, Bent B, McClain MT, et al. Assessment of the feasibility of using noninvasive wearable biometric monitoring sensors to detect influenza and the common cold before symptom onset. *JAMA Netw Open.* 2021;4(9):e2128534. doi:10.1001/jamanetworkopen.2021.28534

eTable 1. Features Used in Random Forest Models
eFigure 1. Feature Sets for Every Model
eFigure 2. Confusion Matrices for Best Performing Model Across Viral Challenges and Infection Status Comparisons
eFigure 3. Confusion Matrices for Best Performing Model Across Viral Challenges and Infection SeverityComparisons
eFigure 4. Relative Feature Importance for Best Performing Model for Each Viral Challenge, Infection Status Grouping, and Infection Severity Grouping
eTable 2. Racial Demographics, Sex, and Median Age Across Infection Severity Groups for H1N1Influenza Viral Challenge and Rhinovirus Viral Challenge
eTable 3. Mean Accuracy, Precision, Sensitivity, F1-Score, AUC of Every Infection Status Model TestedAcross Viral Challenges, Number of Hours Post-Inoculation, and Infection Severity Comparisons
eTable 4. Mean Accuracy, Precision, Sensitivity, Specificity, F1-Score, AUC of Every Infection Severity Model Tested Across Individual Viral Challenges, Number of Hours Post-Inoculation, and Infection Severity Comparisons
eTable 5. Mean Accuracy, Precision, Sensitivity, Specificity, F1-Score, AUC of Every Infection Severity Model Tested Across Combined Viral Challenges, Number of Hours Post-Inoculation, and Infection Severity Comparisons

This supplementary material has been provided by the authors to give readers additional information about their work.

© 2021 Grzesiak E et al. *JAMA Network Open.*

Feature Name	Sensor	Definition	Calculation	Final Feature
Median RHR	HR, ACC	Median Resting Heart Rate	HR when: 5 min weighted rolling ACC window < resting threshold	median(RHR) - median(RHR)$_{baseline}$
Median rTemp	Temp, ACC	Median Resting Temperature	Temp when: 5 min weighted rolling ACC window < resting threshold	median(rTemp) - median(rTemp)$_{baseline}$
Median ACC	ACC	Median Root Mean Squared 3-axis Accelerometer	$sqrt[ACC_x^2 + ACC_y^2 + ACC_z^2]$	median(ACC) - median(ACC)$_{baseline}$
Mean HRV		Mean Heart Rate Variability	mean(RR)	mean(rolling 5 min mean HRV) - mean(rolling 5 min mean HRV)$_{baseline}$
Median HRV		Median Heart Rate Variability	median(RR)	mean(rolling 5 min median HRV) - mean(rolling 5 min median HRV)$_{baseline}$
rMSSD		Root Mean Square of Successive RR Interval Differences	$sqrt[mean((RR_{i+1} - RR_i)^2)]$	mean(rolling 5 min rMSSD) - mean(rolling 5 min rMSSD)$_{baseline}$
NN50	IBI	Number of Successive RR (w/ Filtering, NN) Intervals That Differ by More Than 50 ms	$sum[\|RR_{i+1} - RR_i\| > 50$ ms]	mean(rolling 5 min NN50) - mean(rolling 5 min NN50)$_{baseline}$
pNN50		% of NN Intervals That Differ by More Than 50 ms	(NN50 x 100) / N	mean(rolling 5 min median pNN50) - mean(rolling 5 min median pNN50)$_{baseline}$
SDNN		Standard Deviation of NN Intervals	std[NN50]	mean(rolling 5 min median SDNN) - mean(rolling 5 min median SDNN)$_{baseline}$

eTable 1. Features Used in Random Forest Models. Feature names, definitions, sensors they were derivedfrom, and calculations.

© 2021 Grzesiak E et al. *JAMA Network*

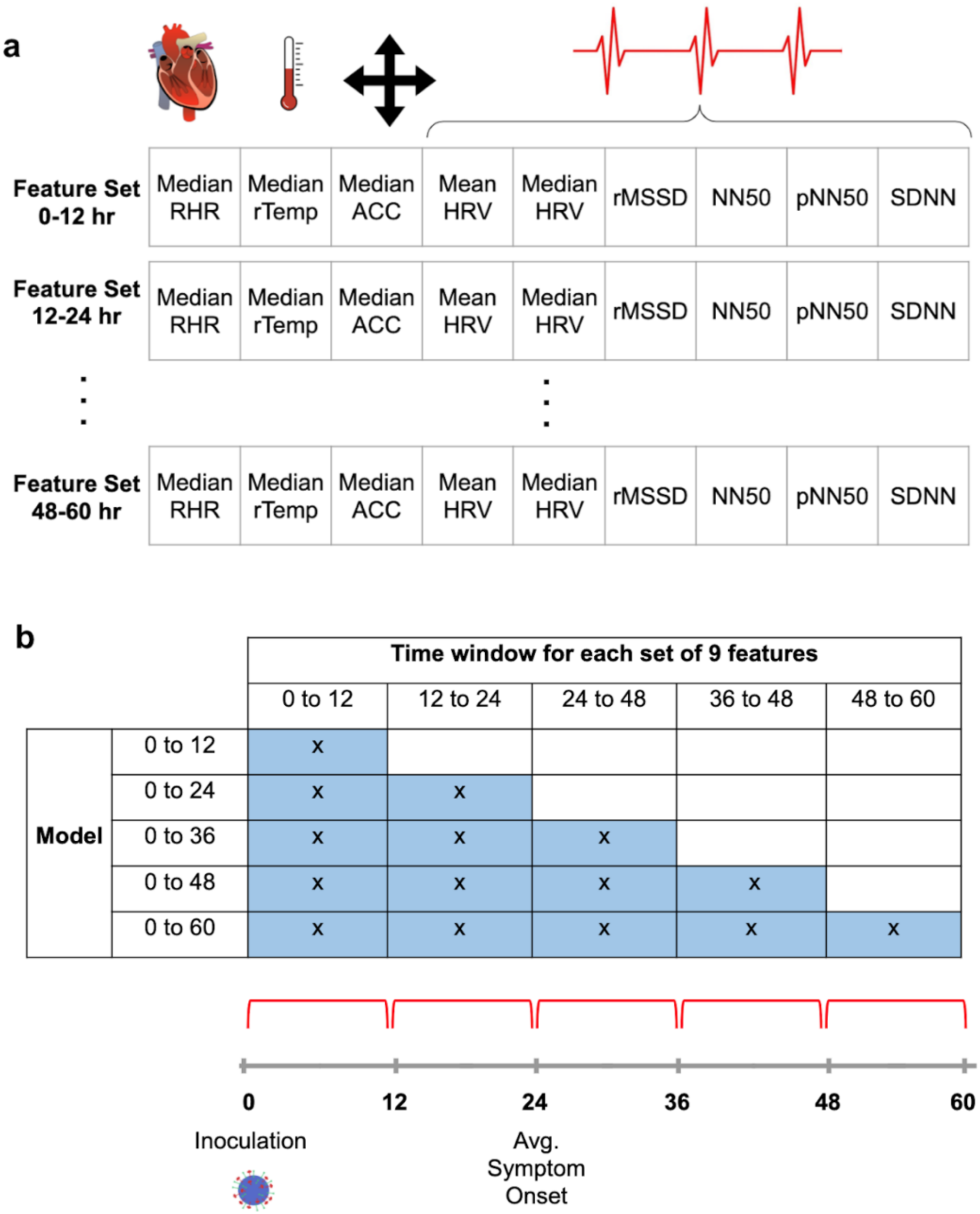

eFigure 1. Feature Sets for Every Model. We used the feature set shown in **a** for each model shown in **b**.The same 9 features are calculated per 12 hr window post-inoculation. Feature sets are only included in models that cover the same time periods.

© 2021 Grzesiak E et al. *JAMA Network*

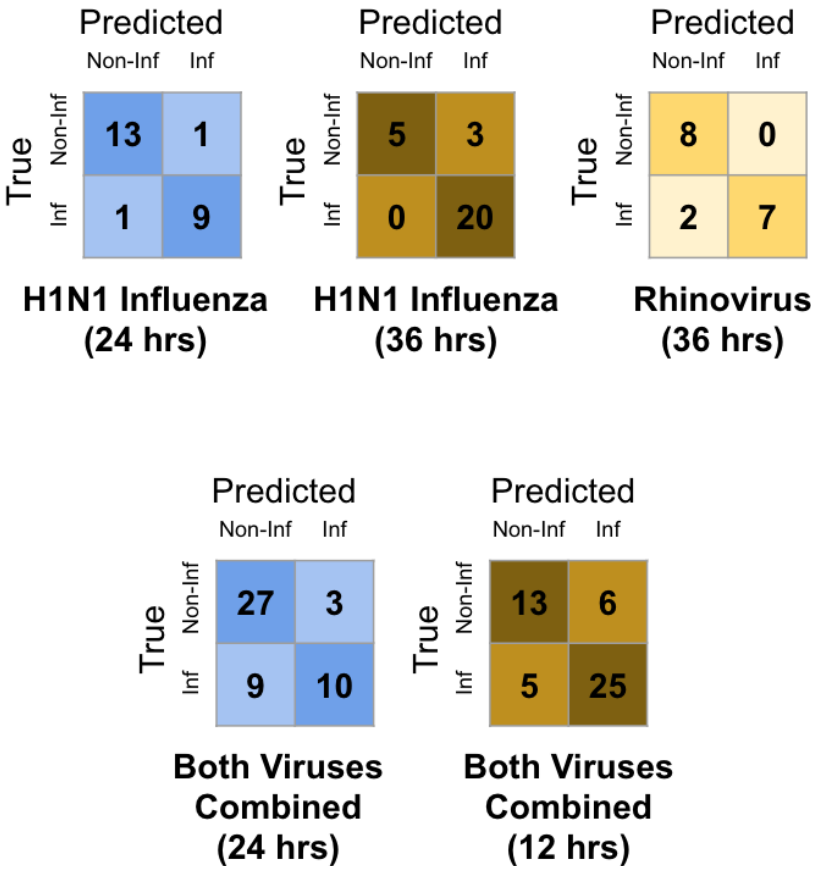

eFigure 2. Confusion Matrices for Best Performing Model Across Viral Challenges and Infection Status Comparisons. Number of hours post-inoculation of best performance is noted per matrix.

© 2021 Grzesiak E et al. *JAMA Network*

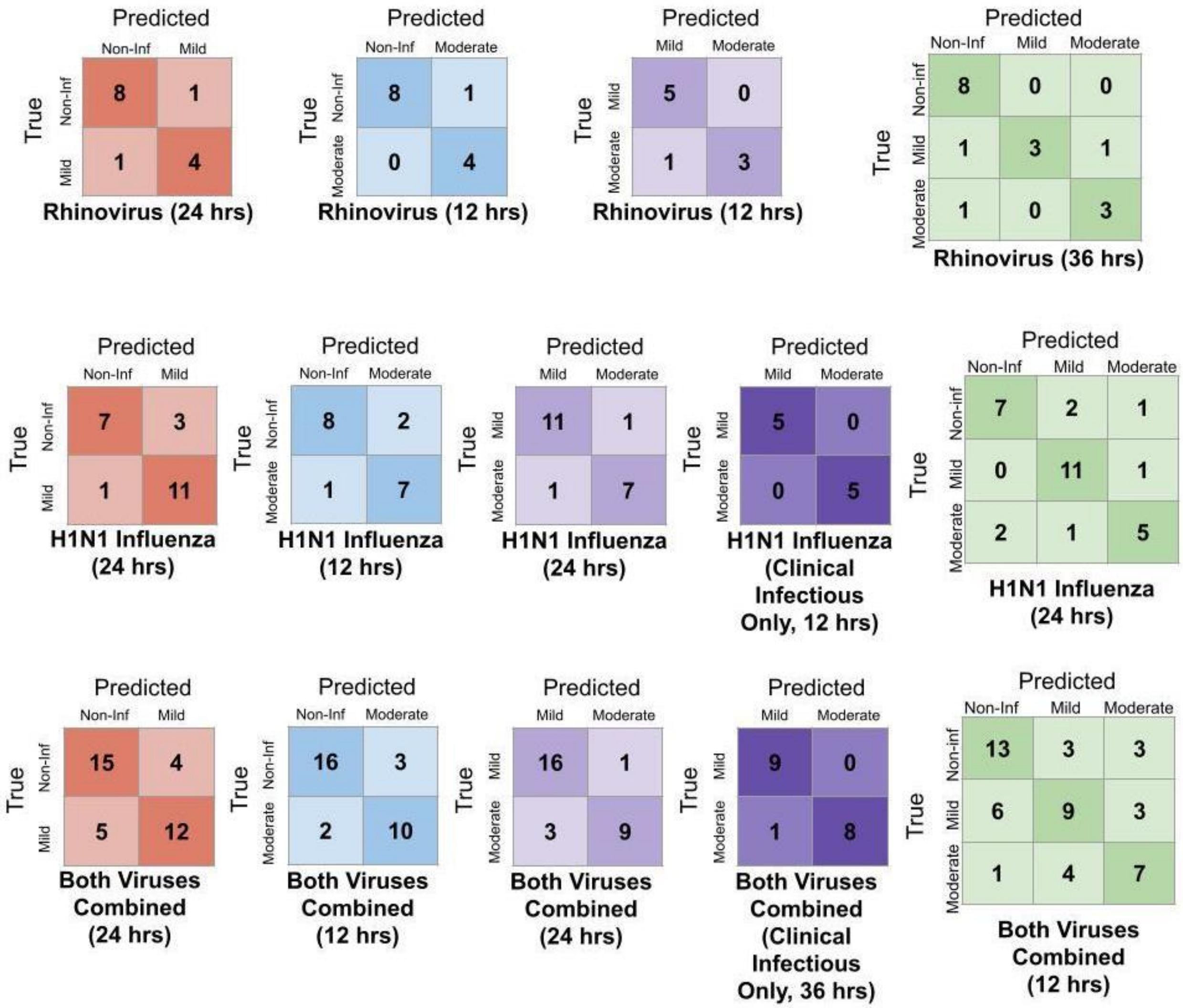

eFigure 3. Confusion Matrices for Best Performing Model Across Viral Challenges and Infection Severity Comparisons. Number of hours post-inoculation of best performance is noted per matrix.

© 2021 Grzesiak E et al. *JAMA Network*

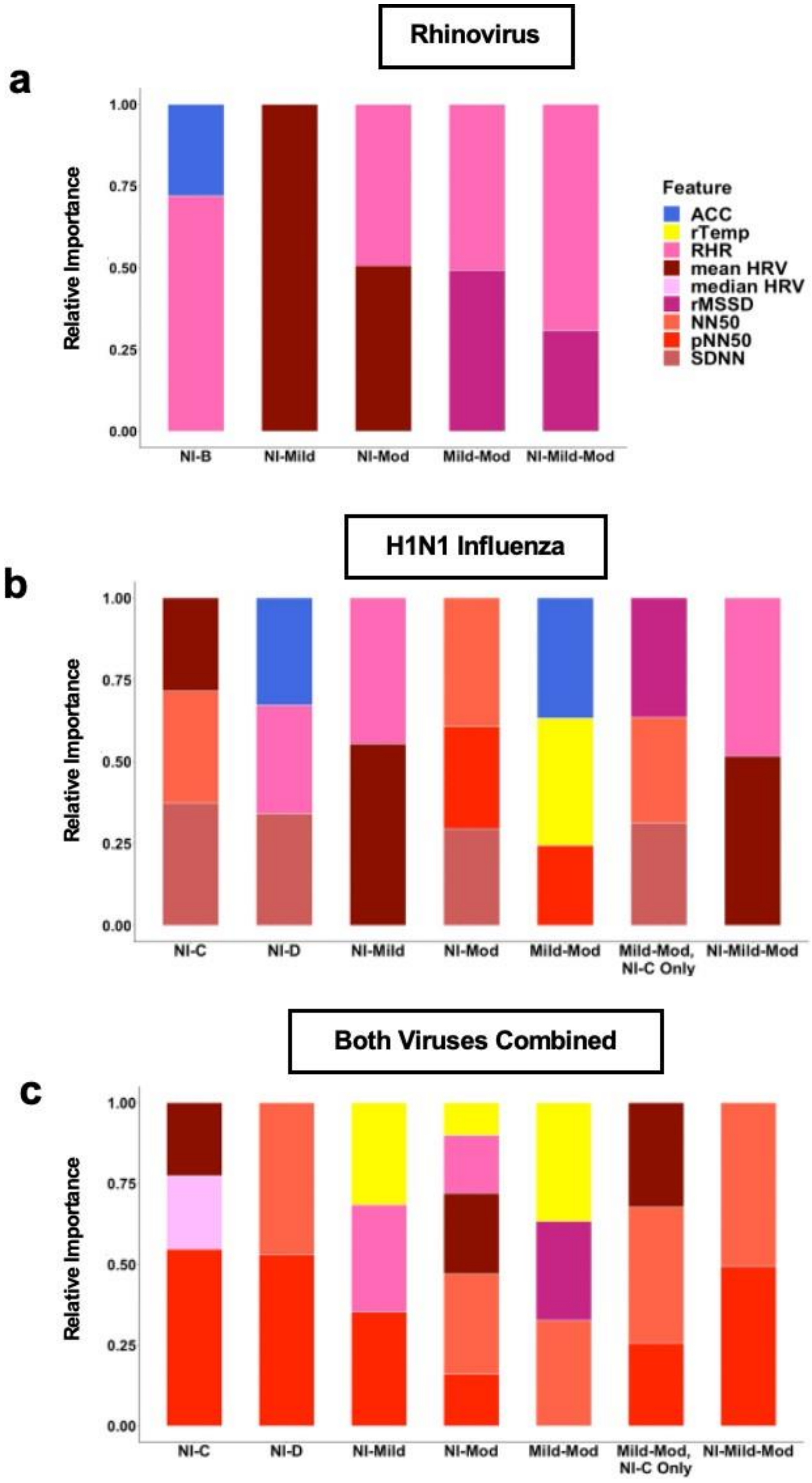

eFigure 4. Relative Feature Importance for Best Performing Model for Each Viral Challenge, Infection StatusGrouping, and Infection Severity Grouping

© 2021 Grzesiak E et al. *JAMA Network*

a

Influenza		Non-Inf	Mild	Moderate	Total
**Median Age (range)**		40 (26-55)	32 (18-55)	33 (23-53)	37 (18-55)
**Sex (N)**	**Male**	9	10	8	27
	**Female**	1	9	2	12
**Race (N)**	**White**	7	11	7	25
	**Black**	0	2	0	2
	**Asian**	2	3	1	6
	**Other**	1	3	2	6

b

Rhinovirus		Non-Inf	Mild	Moderate	Total
**Median Age (range)**		21 (20-24)	20 (20-21)	22 (21-33)	21 (20-33)
**Sex (N)**	**Male**	7	3	1	11
	**Female**	2	2	3	7
**Race (N)**	**White**	7	4	3	14
	**Black**	2	0	0	2
	**Asian**	0	1	1	2

eTable 2. Racial Demographics, Sex, and Median Age Across Infection Severity Groups for **a** H1N1 Influenza Viral Challenge and **b** Rhinovirus Viral Challenge.

© 2021 Grzesiak E et al. *JAMA Network*

Model	Time PI	Accuracy [SD] (%)	Precision (%)	Sensitivity (%)	Specificity (%)	F1-Score (%)	AUC [95% CI]
**flu-infected, clinical**	0-12	79 [41]	72	80	79	76	0.68 [0.46, 0.89]
**flu-infected, data-driven**	0-12	77 [42]	79	90	50	84	0.62 [0.41, 0.84]
**RV-infected, both**	0-12	78 [42]	78	78	78	78	0.77 [0.54, 0.99]
**combined-infected, data-driven**	0-12	78 [41]	81	83	68	82	0.66 [0.50, 0.82]
**combined-infected, clinical**	0-12	69 [46]	69	58	78	63	0.55 [0.37, 0.73]
**flu-infected, clinical**	0-24	92 [27]	90	90	93	90	0.85 [0.70, 1.00]
**flu-infected, data-driven**	0-24	83 [37]	86	90	70	88	0.82 [0.64, 0.99]
**RV-infected, both**	0-24	78 [42]	78	78	78	78	0.77 [0.54, 0.99]
**combined-infected, data-driven**	0-24	78 [41]	81	83	68	82	0.66 [0.50, 0.82]
**combined-infected, clinical**	0-24	76 [43]	76	68	83	72	0.75 [0.60, 0.90]
**flu-infected, clinical**	0-36	92 [27]	90	90	93	90	0.85 [0.70, 1.00]
**flu-infected, data-driven**	0-36	89 [31]	87	100	63	93	0.84 [0.66, 1.00]
**RV-infected, both**	0-36	88 [32]	100	78	100	88	0.96 [0.85, 1.00]
**combined-infected, data-driven**	0-36	78 [41]	81	83	68	82	0.66 [0.50, 0.82]
**combined-infected, clinical**	0-36	76 [43]	76	68	83	72	0.75 [0.60, 0.90]

eTable 3. Mean Accuracy, Precision, Sensitivity, F1-Score, AUC of Every Infection Status Model Tested Across Viral Challenges, Number of Hours Post-Inoculation, and Infection Severity Comparisons

© 2021 Grzesiak E et al. *JAMA Network*

Model	Time PI	Accuracy [SD] (%)	Precision (%)	Sensitivity (%)	Specificity (%)	F1-Score	AUC [95% CI]
**flu-asymptomatic-mild**	0-12	70 [46]	75	69	70	72	0.72 [0.51, 0.94]
**flu-asymptomatic-moderate**	0-12	83 [37]	78	88	80	82	0.88 [0.71, 1.00]
**flu-mild-moderate**	0-12	81 [39]	75	75	85	75	0.86 [0.69, 1.00]
**flu-mild-moderate (Infected Clinical Only)**	0-12	100 [0]	100	100	100	100	1.00 [1.00, 1.00]
**flu-asymptomatic-mild-moderate**	0-12	52 [50]	53	52	75	52	--
**RV-asymptomatic-mild**	0-12	71 [45]	80	71	100	65	0.33 [0.02, 0.65]
**RV-asymptomatic-moderate**	0-12	92 [27]	80	100	89	89	1.00 [1.00, 1.00]
**RV-mild-moderate**	0-12	89 [31]	100	75	100	86	0.95 [0.79, 1.00]
**RV-asymptomatic-mild-moderate**	0-12	67 [47]	66	67	77	66	--
**flu-asymptomatic-mild**	0-24	82 [39]	79	92	70	85	0.75 [0.53, 0.96]
**flu-asymptomatic-moderate**	0-24	83 [37]	78	88	80	82	0.88 [0.71, 1.00]
**flu-mild-moderate**	0-24	90 [30]	88	88	92	88	0.88 [0.72, 1.00]
**flu-asymptomatic-mild-moderate**	0-24	77 [42]	76	77	88	76	--
**RV-asymptomatic-mild**	0-24	86 [35]	80	80	89	80	0.71 [0.43, 0.99]
**RV-asymptomatic-moderate**	0-24	92 [27]	80	100	89	89	1.00 [1.00, 1.00]
**RV-mild-moderate**	0-24	89 [31]	1	75	100	86	0.95 [0.79, 1.00]
**RV-asymptomatic-mild-moderate**	0-24	67 [47]	66	67	77	66	--
**RV-asymptomatic-mild-moderate**	0-36	82 [38]	85	82	88	82	--

eTable 4. Mean Accuracy, Precision, Sensitivity, Specificity, F1-Score, AUC of Every Infection Severity Model Tested Across Individual Viral Challenges, Number of Hours Post-Inoculation, and Infection SeverityComparisons

© 2021 Grzesiak E et al. *JAMA Network*

Model	Time PI	Accuracy [SD] (%)	Precision (%)	Sensitivity (%)	Specificity (%)	F1-Score	AUC [95% CI]
**combined-asymptomatic-mild**	0-12	62 [49]	61	61	63	61	0.64 [0.46, 0.82]
**combined -asymptomatic-moderate**	0-12	84 [37]	77	83	84	80	0.78 [0.61, 0.94]
**combined-mild-moderate**	0-12	70 [46]	67	50	83	57	0.65 [0.45, 0.85]
**combined-mild-moderate (Infected Clinical Only)**	0-12	79 [41]	72	89	70	80	0.76 [0.53, 0.98]
**combined-asymptomatic-mild-moderate**	0-12	59 [49]	59	59	79	59	--
**combined-asymptomatic-mild**	0-24	75 [43]	71	73	79	70	0.70 [0.52, 0.87]
**combined -asymptomatic-moderate**	0-24	84 [37]	77	83	84	80	0.78 [0.61, 0.94]
**combined-mild-moderate**	0-24	86 [34]	90	75	94	82	0.91 [0.80, 1.00]
**combined-mild-moderate (Infected Clinical Only)**	0-24	89 [31]	89	89	89	89	0.93 [0.79, 1.00]
**combined-asymptomatic-mild-moderate**	0-24	59 [49]	59	59	79	59	--
**combined-asymptomatic-mild**	0-36	75 [43]	71	73	79	70	0.70 [0.52, 0.87]
**combined -asymptomatic-moderate**	0-36	84 [37]	77	83	84	80	0.78 [0.61, 0.94]
**combined-mild-moderate**	0-36	86 [34]	90	75	94	82	0.91 [0.80, 1.00]
**combined-mild-moderate (Infected Clinical Only)**	0-36	94 [23]	100	89	100	94	0.94 [0.82, 1.00]
**combined-asymptomatic-mild-moderate**	0-24	59 [49]	59	59	79	59	--

eTable 5. Mean Accuracy, Precision, Sensitivity, Specificity, F1-Score, AUC of Every Infection Severity Model Tested Across Combined Viral Challenges, Number of Hours Post-Inoculation, and Infection Severity Comparisons

© 2021 Grzesiak E et al. *JAMA Network*
